# Exploratory study of the effects of intra-uterine growth retardation and neonatal energy supplementation of low birth-weight piglets on their post-weaning cognitive abilities

**DOI:** 10.1007/s10071-019-01251-8

**Published:** 2019-02-28

**Authors:** Océane Schmitt, Keelin O’Driscoll, Emma M. Baxter

**Affiliations:** 1 Pig Development Department, Teagasc Animal and Grassland Research and Innovation Centre, Moorepark, Fermoy, CoCork, Ireland; 2 Department of Animal Production, Easter Bush Veterinary Centre, Royal (Dick) School of Veterinary Studies, The University of Edinburgh, Easter Bush Campus, EH25 9RG Midlothian, UK; 3 Animal Behaviour and Welfare Team, Animal and Veterinary Sciences Research Group, SRUC, West Mains Road, EH9 3JG Edinburgh, UK

**Keywords:** Pigs, Habituation, Learning, Memory, IUGR, Energy supplementation

## Abstract

The present study investigated the effects of intra-uterine growth retardation (IUGR, score 0–3; i.e., “normal” to “severe”) level at birth, and the effects of neonatal energy supplementation (dosed with 2 ml of coconut oil, commercial product or water, or sham-dosed), on post-weaning cognitive abilities of low birth-weight piglets (< 1.1 kg). In total, 184 piglets were recruited at weaning (27 ± 0.1 days) for habituation to the test procedures, and were either tested for spatial learning and memory in a T-maze (*n* = 42; 37 ± 0.5 days) or for short-term memory in a spontaneous object recognition task (SORT; *n* = 47; 41 ± 0.3 days). Neonatal supplementation did not affect performances of pigs in the T-maze task or SORT. IUGR3 pigs tended to be faster to enter the reward arm and to obtain the reward in the reversal step of the T-Maze task, suggesting a better learning flexibility, compared to IUGR1 (entry *t*_72.8_=2.9, *P* = 0.024; reward *t*_80_ = 3.28, *P* = 0.008) and IUGR2 (entry *t*_70.3_=2.5, *P* = 0.068; reward *t*_73.9_ = 2.77, *P* = 0.034) pigs. However, a higher percentage of IUGR1 pigs tended to approach the novel object first (DSCF-value = 3.07; *P* = 0.076) and to interact with it more (*t*_40_ = 2.19, *P* = 0.085), relative to IGUR3 pigs. IUGR1 pigs showed a strong preference for the novel object, as they had a greater percentage time difference interacting with the objects when the novel object was presented (*t*_81_ = − 3.41, *P* = 0.013). In conclusion, some low birth-weight piglets are able to perform a spatial task and an object recognition test, but performances in these tests may be modulated by IUGR level.

## Introduction

The characteristics of piglets at birth can influence their cognitive abilities. The effect of birth-weight is most widely studied; however, there are contradictory results in the literature. Some studies demonstrated that low birth-weight piglets (maximum of all studies: 1.05 kg birth-weight) had poorer cognitive abilities than normal birth-weight piglets (average in studies 1.45 kg birth-weight) (Gieling et al. 2012; Radlowski et al. 2014; Roelofs et al. 2018), whereas there is some evidence of no difference (Antonides et al. 2015a). Vazquez-Gomez et al. (2016) recently found that cognitive abilities of pigs might be modulated by both birth-weight and gender. Indeed, low and normal birth-weight females did not differ in concentrations of catecholamine neurotransmitters, suggesting similar cognition abilities; whereas males did, suggesting impaired cognitive abilities in low birth-weight males compared to normal birth-weight counterparts (Vazquez-Gomez et al. 2016). However, to date, we know of no studies that investigated the validity of these findings in cognitive tests. However, the study by Roelofs et al. (2017) showed that normal birth-weight female piglets performed better than males in a hole-board task.

Prior to birth, low birth-weight human infants initiate a circulatory redistribution process called the ‘brain-sparing effect’, which is an adaptation to cope with placental insufficiency, and aims to ensure normal development of the brain by maintaining the oxygen supply (Roza et al. 2008). The ‘brain-sparing effect’ is often characterised by an asymmetric growth of vital organs in the foetus, reflected by a low middle cerebral artery pulsatility index, which has also been observed in piglets (e.g., Chevaux et al. 2010; Hales et al. 2013). This process might also have an influence on the cognitive abilities of low birth-weight piglets. The level of intra-uterine growth retardation (IUGR), which is associated with low birth-weight piglets, could also affect their performance. Piglets born with low birth-weight do not necessarily suffer from IUGR, and the level of IUGR can vary amongst piglets (Chevaux et al. 2010). Thus the level of IUGR should be taken into consideration when assessing cognitive development of low birth-weight piglets. Piglets with different levels of IUGR at birth could vary in cognitive development, and thus differ in cognitive abilities post-weaning. To date there are no studies looking at the effect of severity of IUGR on cognitive abilities of piglets.

Hole-board tasks are widely used to assess the spatial learning and memory abilities of pigs (Gieling et al. 2012; Radlowski et al. 2014; Roelofs et al. 2018). However, such tests require complex equipment and lengthy training of the pigs. A simpler test, validated for testing spontaneous trial-unique memory in pigs (Moustgaard et al. 2002), is the Spontaneous Object Recognition Test (SORT; Gieling et al. 2011). In this test a pig is initially exposed to two identical objects in a test pen. The test pig is then re-introduced to the pen after a short period of time, during which one of the objects is replaced with a novel object. The test assesses the animals’ object discrimination and short-term memory capabilities (Gieling et al. 2011). Long-term memory, spatial learning, and learning flexibility can be assessed using a T-maze task, validated by Elmore et al. (2012), where a pig has to retrieve a reward in a T-shaped maze using visual cues. The pig initially learns the location of a reward in one arm of a T-maze, as indicated by extra-maze cues. The flexibility of learning is assessed during a reversal phase, where animals are asked to switch from the learned reward arm to an opposing arm, to obtain the reward. This test should be achievable by low birth-weight piglets as they do not differ from normal birth-weight pigs in food motivation (van Eck et al. 2016). Indeed, both low birth-weight (0.7–1.0 kg) and normal birth-weight piglets (1.3–1.6 kg) successfully learned the T-maze spatial task, but low birth-weight piglets took a day longer to reach success criterion (Radlowski et al. 2014). In their validation study, Elmore et al. (2012) suggested that training success of the pigs in the T-maze task might be influenced by nutritional deficits.

Diet and nutritional status can also influence the cognitive abilities of animals (Bushby et al. 2018). For instance, under-nutrition affects sheep’s emotional reactivity and cognitive flexibility (Erhard et al. 2004), and iron deficits in piglets also impair their cognitive performances in hole-board tasks (Antonides et al. 2015b) and T-maze tasks (Rytych et al. 2012). Since energy supplementation of neonatal low birth-weight piglets should improve their survival and growth (Declerck et al. 2016; Muns et al. 2017), it could be hypothesised that it would help their brain development (e.g., promoting the brain-sparing effect). Fat-based energy products containing medium-chain fatty acids are easily absorbed and used by piglets (Heo et al. 2002), thereby enhancing their energy status (Heo et al. 2002), which should consequently promote their thermoregulatory abilities (Herpin et al. 2002). Contrarily, dosing piglets with water would fill their stomachs and give them a feeling of satiety without providing energy (negative control; Schmitt et al., submitted), which might result in a delayed colostrum intake and a lower energetic status, as suggested by the drop in blood glucose content between dosing and 27 h post-partum (Schmitt et al., submitted). The aim of this study was to compare the cognitive abilities of low birth-weight piglets with different levels of IUGR at birth, and to determine if provision of an energy supplement (coconut oil or commercial product) or not (water or sham-dosed) at birth would affect them. The effect of gender was also investigated. It was hypothesised that (1) low birth-weight piglets are capable of learning the T-Maze and SOR tasks; (2) piglets with no (or low) IUGR levels (score 0 or 1) would have better cognitive abilities than piglets with high IUGR levels (score 3); (3) piglets which received energy at birth would have enhanced cognitive abilities, compared to piglets which did not; and (4) female piglets would have better cognitive abilities than males.

## Materials and methods

### Ethical approval

Ethical approval for this study was granted by Teagasc Animal Ethics Committee (application TAEC133/2016). The experiment was carried out in accordance with the Irish legislation (SI no. 543/2012) and the EU Directive 2010/63/EU for animal experiments. At the end of the experiment, animals were returned to the commercial herd.

### Animals and experimental design

This experiment was conducted in the Teagasc Moorepark Research Centre, Co. Cork, Ireland. A total of 184 piglets from 58 litters were recruited at weaning (27 ± 0.1 days) to undergo habituation to the testing procedures (see below). Piglets were allowed six sessions of each habituation step (as described below) to be selected for testing. Only 89 low birth-weight piglets from 43 litters passed the habituation process and were tested in one of the two cognition tasks (see below). The overall male/female ratio was 0.98 (44 males and 45 females): 0.91 for the T-Maze test and 1.04 for the SORT. These pigs were recruited over five batches of farrowing/weaning, two of which were recruited for the SORT and three for the T-maze task. Genetic background of the piglets was Large White × Duroc.

Piglets were born in conventional farrowing pens (250 × 181 cm) containing a sow crate (225 × 60 cm), a heat pad (155 × 37 cm; 2/3 covered), and a water cup and a feeder for piglets. Piglets used in this study were part of a larger experiment looking at the effects of neonatal energy supplementation on piglets’ performance and vitality (Schmitt et al., submitted). Details of the management of the piglets pre-weaning can be found in that paper. In brief, piglets were tail docked at one day post-partum, following veterinary authorisation, but were not teeth-clipped or castrated. Piglets also received an injection of iron (Gleptosil®, Ceva) at four days post-partum, and they were vaccinated against Porcine Circovirus type 2 (Porcilis® PCV ID, MSD) on the day of weaning (27 ± 0.1 days of age).

Within 3 h of birth, piglets which weighed < 1.1 kg were recruited and were randomly (within litter) assigned to one of the following four treatments (applied at 3 h post-partum): (1) sham-dosed (S, *n* = 24), (2) dosed with 2 ml of coconut oil (CO, *n* = 24; 72 kJ), (3) a commercial energy product (CP, *n* = 20; 71 kJ) or (4) water (W, *n* = 21; 0 kJ). At this time piglets were scored for their level of intra-uterine growth retardation (IUGR) following the method by Hales et al. (2013). The presence/absence of a dolphin-shaped head, bulging eyes and wrinkles on the snout were recorded. IUGR scores ranged from 0 when none of the IUGR characteristics were present on the piglet, to 3 when all three IUGR characteristics were present on the piglet.

Piglets were weaned at 27 ± 0.1 days into pens of 12 piglets, with neonatal supplementation and gender balanced in each pen. Over representation of the same litter was avoided as much as possible. One week after weaning, all pigs underwent a habituation protocol prior to recruitment for one of the two tests (details below). Pigs failing the habituation protocol were not considered further in the study. In total, 42 piglets (S = 12 piglets, W = 10 piglets, CP = 8 piglets and CO = 12 piglets/IUGR0 = 6 piglets, IUGR1 = 15 piglets, IUGR2 = 14 piglets, IUGR 3 = 7 piglets) were tested for spatial learning in the T-maze task and 47 piglets (S = 12 piglets, W = 11 piglets, CP = 12 piglets and CO = 12 piglets/IUGR1 = 17 piglets, IUGR2 = 17 piglets, IUGR 3 = 13 piglets) were tested for spontaneous object recognition in the SORT. Given that there was no IUGR0 piglet tested in the SORT, there are no results for this category of piglets.

### Nutrition

Details of the sow diets and creep feed given to piglets during lactation can be found in Schmitt et al. (submitted). Creep feed was provided from 10 days of age. The post-weaning diet given to the piglets contained 87.6% dry matter, 18.5% protein, 6.7% fat, and had an energetic value of 10.3 MJ/kg (net energy).

### Housing

Piglets were housed in groups of 12 in pens (250 × 197 cm) equipped with a canopy (250 × 72 cm, placed 84 cm above the ground) which provided thermal comfort to the pigs. Room temperature was maintained at 27 °C for the first two weeks post-weaning, and then temperature decreased by 1 °C every week. They were fed ad libitum through a feeder (28 × 28 cm) which allowed only one pig to feed at a time. There was a plastic pad (65 × 56 cm) in front of the feeder to limit knee injuries and food wastage. Two nipple drinkers for ad libitum water consumption were accessible in the pen: one was fitted in the feeder and the second one was placed against a pen wall. A rubber floor toy was given to pigs in each pen as enrichment (Easyfix® LUNA 117; Easyfix, Ballinasloe, Ireland).

In the test rooms, minimum and maximum room temperatures were recorded once daily at 1700 h. In the room where the T-maze was fitted, temperature ranged from 21.4 ± 0.12 to 22.7 ± 0.11 °C. In the room where pigs were tested for SORT, temperature ranged from 22.6 ± 0.16 to 23.7 ± 0.08 °C.

### T-Maze test

#### Apparatus

**Fig. 1:**
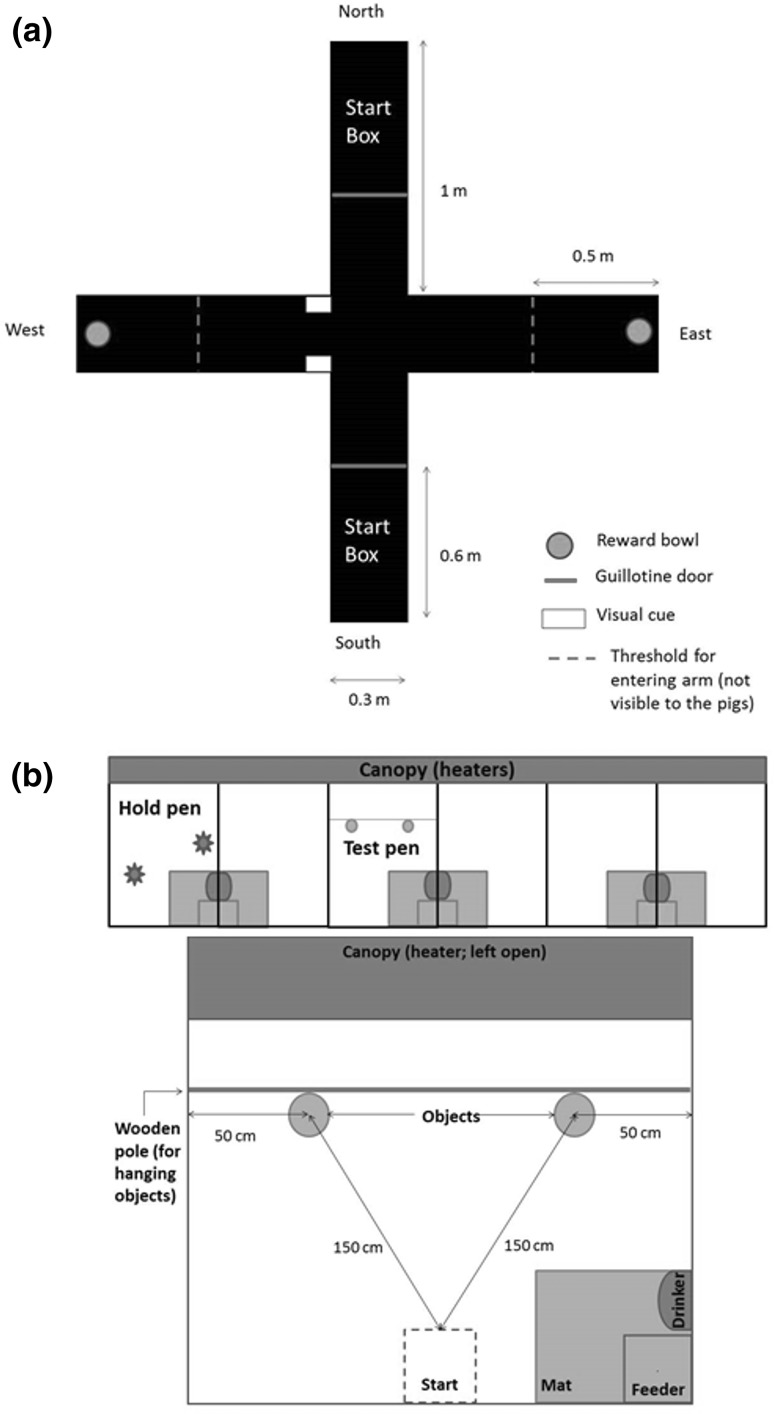
Schematic representation of the experimental set-up. **a** T-Maze task. The apparatus was fitted in a room (416.5 × 482.6 cm) with slatted concrete floor and grey walls. Black rubber mats were placed under the apparatus to prevent pigs from getting cold. The arm opposite to the assigned start arm was blocked with the guillotine door. **b** Spontaneous Object Recognition Test pen design. The pen (210 × 195 cm) was situated in an isolated room which contained six identical pens, one of which was the hold pen (two pens apart, on the left-hand side). The two objects were hung approximately 0.15 m above the ground, to be in the pig line of sight. The feeder was empty to avoid distraction. Blue star shapes in the hold pen represent enrichment objects

The apparatus was a double T-maze (Fig. 1a) located in a room with concrete slatted floor and grey walls (416.5 × 482.6 cm). Black solid rubber mats were placed under the apparatus to prevent pigs from getting cold and injuries to the feet. Two arms contained a start box (North and South) and the two other arms were choice arms (East and West), to ensure that the pigs used an allocentric mechanism (rather than an egocentric mechanism) to locate the food reward. Extra-maze visual cues consisted of white adhesive stripes displayed on the walls at the entry of the West arm. Both choice arms contained a blue plastic bowl (24 cm diameter, 10 cm high) containing a food reward, one of which was covered with a metal mesh to prevent the pig from accessing the reward. This ensured that pigs were not able to locate the reward using olfactory cues. Both arms were rewarded during habituation and only one arm was rewarded during training and testing.

#### Habituation to experimental procedure

At approximately 29 ± 0.3 days, pigs were habituated to the procedure in four stages: (1) human handling, (2) transport in a wheelbarrow bedded with straw (group and alone), (3) placement in the apparatus (group and alone), and (4) test procedure (e.g., opening and closing of doors). Habituation sessions were conducted morning and afternoon to allow the pigs to be tested at any time of day thereafter. At any stage of habituation, if a pig showed a panic reaction (e.g., repeated attempts to escape, loud distress-like squealing, and repeated defecations within or over sessions) it was removed from the study. Pigs were also removed if they did not habituate before the sixth session of any of the four habituation stages.

#### Training and testing

Pigs started the training sessions at approximately 37 ± 0.5 days. Pig behaviour in the apparatus was recorded continuously using a handheld device (Psion Workabout Pro™ 3, Motorola Inc., Schaumburg, Illinois, USA) equipped with the software The Observer XT (Noldus, Wageningen; The Netherlands). A radio was played to minimise the effects of unpredictable noises on the pigs’ performance in the task. The start point (North or South) was randomly assigned between trials using a randomisation schedule. If a pig soiled the apparatus during a session, it was cleaned with water before the next pig was tested. The apparatus was thoroughly washed daily with water after the last session. Each training and testing session consisted of ten trials of 60 s (s). Food rewards were one chocolate peanut (Milk chocolate American peanuts, Tesco© Stores Ltd.) and three honey coated puff cereals (Crownfield, Lidl Stiftung & Co.). For each trial, the arm opposite to the start point was blocked using a guillotine door, so the test pig could only enter one of the two choice arms (i.e., it could not enter the opposing start arm of the apparatus). A trial was considered successful if the pig entered the reward arm, whether or not the reward was consumed. The pig was considered to have entered the arm if both forelegs passed a line drawn at 0.5 m from the reward bowl. At the end of a trial, the test pig was gently guided by the experimenter to the next starting box. At the end of a session, the test pig was lifted from the apparatus, placed in the wheelbarrow and returned to the home pen.

For training sessions, each pig was randomly assigned a choice arm (East or West) which would contain the accessible reward. Training was done in two steps: (1) the pig could make a mistake and continue exploring the maze to retrieve the reward within the 60 s of the trial starting; (2) The pig was only permitted one attempt to locate the reward, and the trial was stopped if it failed to enter the reward arm. Training steps were considered complete if the pig reached the success criterion of 80% (i.e., eight out of ten trials were successful). Therefore, in training step 1 successful pigs obtained the reward in eight out of ten trials, independent of which arm they entered first; but in training step 2 successful pigs had to enter the reward arm first in eight out of ten trials. The trial could be stopped before the 60 s when the pig being tested finished consuming the reward, or entered the non-reward arm in training step 2 and reversal. If by the fifth training session pigs did not reach the success criterion, they were considered a “non-learner” and training was stopped. A pig was removed from the experiment if it was ill or if it lied down or stayed still in the apparatus during 50% of the trials over two consecutive days. Trials where the pig failed to enter any arm within 60 s were considered “non-compliant”.

When a pig completed training the testing phase began. This consisted of a “reversal phase”, where the reward arm for each test pig was reversed. Test sessions followed the same procedure as training sessions, and the pig only had one attempt permitted per trial. The reversal phase was stopped if the pig reached the success criterion (80% of trials successful) or after three sessions (maximum allowed).

### Spontaneous Object Recognition Test (SORT)

#### Apparatus

The apparatus for the SORT consisted of a test pen (Fig. 1b), where objects were presented to the test pig, and a holding pen, where the test pig was placed during retention time (i.e., between test sessions). Both pens were located in the same room, had the same dimensions (210 × 195 cm), and were equipped with a canopy, a feeder and a nipple drinker (Fig. 1b). The holding pen was enriched with two floor toys (EasyFix^®^ LUNA 117; Easyfix, Ballinasloe, Ireland), a hessian bag attached to a wall and a handful of straw on the floor. In the test pen, test objects (see description below) were suspended from a wooden bar with orange plastic ropes, 50 cm from the side walls and 15 cm from the ground (i.e., at pig eye level).

#### Habituation

Pigs started to be habituated to the test procedure at approximately 29 ± 0.3 days. For a week prior to testing, test pigs were habituated to (1) human handling, (2) transport in a wheelbarrow, (3) the holding pen with other pigs present, and (4) being isolated in another pen. The procedure was carried out as described previously.

#### Testing

On the test day (approximately 40 ± 0.3 days), pigs were brought into the holding pen with two companion pigs which were also habituated for the test but not tested on the same day. Thus at any time there were a pair of pigs in the holding pen, which minimised stress due to prolonged social isolation. The SORTs consisted of two sessions. If during session 1 the pig attempted to jump out of the pen, it did not progress to session 2.

During session 1, two identical objects (metal creep feeders, 25 cm diameter × 16 cm high) were presented to the test pig. The objects were suspended at pig’s eye level in the test pen prior to the pigs entering the pen. Pigs remained in the pen for 5 min, after which the pig returned to the holding pen for a 15-min retention period. Following this, the pig was returned to the test pen for session 2. Before session 2, one of the familiar objects was replaced by a novel object (bamboo stick, 5 cm diameter × 40 cm high). The side of the pen in which the novel object was placed was systematically randomised by neonatal supplementation and gender.

Testing sessions were video recorded (no human presence in the room during test) and videos were analysed by a single observer (intra-observer reliability = 95%) using The Observer XT (Noldus, Wageningen, The Netherlands). The latency to approach the objects and the time spent physically interacting with either object were recorded.

### Statistical analyses

Statistical analyses were performed using SAS 9.4 (SAS Inst. Inc., Cary, NC, USA). The experimental unit for the analysis was the pig. General linear models (GLM) and generalised linear mixed models (GLMM) were fitted using the Residual Pseudo Likelihood approximation method. Statistically significant terms were determined when alpha was less than 0.05 and tendencies were determined when alpha was between 0.05 and 0.1. Batch and weaner pen were included as random effects in all models. Neonatal supplementation, IUGR level and gender were included as fixed effects in all models. Side (SORT) and the attributed reward arm (T-maze) were also included in models as fixed effects, except for the analysis of habituation sessions in the T-Maze task. The effects of supplementation and IUGR were investigated separately since the interaction supplementation × IUGR was not significant. For both tests, each session (SORT) or step (T-Maze) was analysed separately; except when researching the effect of session on the time interacting with the familiar object and with both objects (SORT), where models included the repeated effect of session.

The percentage differences in time spent interacting with the objects were calculated as the difference between the percentage of time interacting with the novel object (or object on the side of the novel object in session 1) and the percentage of time interacting with the familiar object (matched for side in session 1). Therefore, positive values reflected preferences for the novel object (session 2) or novel object side (session 1). Durations, latencies (to enter arm or get reward, or to approach the novel object), the proportion of time spent interacting with the objects, and the percentage differences in time spent interacting with the objects were analysed using GLMs. Success rates and number of sessions to complete a step were analysed using GLMMs with Poisson distribution and a log link function. As only three pigs failed to complete the training step 1, the estimated least-square means for success rate were virtually 100% for each category of piglet (IUGR level and neonatal supplementation). Therefore, for better representation of reality, raw means and standard errors are presented in Tables 1 and 2. As very few pigs reached reversal step, the success rate of this step was analysed without the fixed effect of arm and without the random effects of batch and weaner pen, which were making the model too complex for our data. GLMMs without the fixed effect of arm and without the random effects of batch and weaner pen were also used to analyse the rate of non-compliant trials (when the pig failed to enter any arm during the trial), as these events were rather rare.

**Table 1:** Mean (± S.E.) outcomes of the T-maze spatial task

	Sham-dosed	Coconut oil	Commercial product	Water	*F* value	*P* value
Habituation						
Number of pigs	20	21	16	19		
Number of sessions	9.6 ± 1.13	10.7 ± 1.24	10.1 ± 1.26	10.6 ± 1.26	*F*(3,65) = 0.48	0.695
Success rate (%)	62.2 ± 13.20	58.0 ± 13.34	39.1 ± 14.48	52.6 ± 13.98	*F*(3,68) = 0.62	0.603
Training 1						
Number of pigs	12	12	7	10		
Number of sessions	1.8 ± 0.49	1.3 ± 0.37	1.1 ± 0.47	1.3 ± 0.41	*F*(3,32) = 0.54	0.662
Success rate (%)^1^	91.7 ± 8.33	91.7 ± 8.33	100 ± 0.00	90.0 ± 10.00	*F*(3,32) = 0.04	0.988
Latency to enter successful arm (s)	24.2 ± 1.73	28.8 ± 1.93	29.4 ± 3.05	23.4 ± 2.00	*F*(3,133) = 2.88	0.073
Latency to reward (s)	30.0 ± 1.76	33.4 ± 1.89	34.4 ± 3.00	29.6 ± 2.09	*F*(3,122) = 1.12	0.344
Duration of trial (s)	59.1 ± 1.18	58.0 ± 1.30	58.2 ± 2.00	55.3 ± 1.41	*F*(3,174) = 1.79	0.150
Non-compliant trials (%)	12.9 ± 2.75^a^	9.1 ± 2.31	3.8 ± 1.68^b^	7.8 ± 2.37	*F*(3,592) = 3.07	0.027
Training 2						
Number of pigs	11	11	7	9		
Number of sessions	2.4 ± 0.48	2.8 ± 0.52	2.5 ± 0.83	2.0 ± 0.47	*F*(3,1) = 0.54	0.732
Success rate (%)	86.5 ± 10.36	76.5 ± 13.42	65.6 ± 22.8	82.7 ± 12.72	*F*(3,1) = 0.31	0.830
Latency to enter successful arm (s)	17.0 ± 1.53	16.9 ± 1.37	15.2 ± 2.36	17.9 ± 1.81	*F*(3,80.4) = 0.25	0.860
Latency to reward (s)	20.6 ± 1.86	22.5 ± 1.74	20.4 ± 2.72	22.3 ± 2.17	*F*(3,117) = 0.38	0.768
Duration trial (s)	37.5 ± 1.92	39.1 ± 1.84	34.6 ± 2.64	39.8 ± 2.22	*F*(3,202) = 1.03	0.382
Non-compliant trials (%)	6.3 ± 1.41	6.7 ± 1.42	8.6 ± 2.67	5.6 ± 1.69	*F*(3,952) = 0.38	0.767
Reversal						
Number of pigs	9	8	5	7		
Number of sessions	2.6 ± 0.59	2.7 ± 0.61	2.6 ± 0.93	3.0 ± 0.71	*F*(3,1) = 0.1	0.948
Success rate (%)	52.1 ± 19.10	49.2 ± 18.76	52.1 ± 26.69	58.8 ± 20.11	*F*(3,21) = 0.04	0.989
Latency to enter successful arm (s)	10.9 ± 2.75	14.0 ± 2.39	13.9 ± 3.24	15.8 ± 2.49	*F*(3,87.5) = 1.33	0.271
Latency to reward (s)	15.0 ± 2.95	17.9 ± 2.66	18.1 ± 3.46	21.0 ± 2.76	*F*(3,90.1) = 2.02	0.117
Duration trial (s)	29.6 ± 4.24^a^	32.5 ± 4.01	39.4 ± 4.66^b^	37.0 ± 4.05^b^	*F*(3,191) = 4.67	0.004
Non-compliant trials (%)	15.3 ± 2.65	8.1 ± 1.86	15.2 ± 3.71	15.9 ± 2.73	*F*(3,802) = 2.44	0.064

Tested piglets either received a dose of energy (coconut oil or commercial product) or water (water), or were sham-dosed (sham), at 3 h post-partum. Habituation to the experimental procedure started 3 days post-weaning (29 ± 0.3 days) and training started at approximately 37 (± 0.5) days. Training and reversal sessions lasted 60 s. During training 1 sessions, pigs were allowed to enter both choice arms to retrieve the reward (mistake allowed). In training 2 sessions, pigs were only allowed one entry attempt. In the reversal sessions, the reward arm was opposite to the one learned in training sessions, and pigs were allowed only one entry attempt

Superscript letters indicate significant differences between the neonatal supplementations at *P* < 0.05

^1^Numbers presented are the raw values

**Table 2:** Mean (± S.E.) outcomes of the T-maze spatial task

	IUGR0	IUGR1	IUGR2	IUGR3	*F* value	*P* value
Habituation						
Number of pigs	13	28	23	12		
Number of sessions	10.6 ± 1.33	9.9 ± 1.11	10.3 ± 1.18	10.2 ± 1.33	*F*(3,65) = 0.16	0.926
Success rate (%)	44.9 ± 15.94	51.0 ± 12.23	62.5 ± 12.85	53.3 ± 17.11	*F*(3,68) = 0.33	0.803
Training 1						
Number of pigs	6	15	14	6		
Number of sessions	1.3 ± 0.51	1.2 ± 0.34	1.3 ± 0.34	1.6 ± 0.74	*F*(3,32) = 0.1	0.962
Success rate (%)^1^	100.0 ± 0.00	93.3 ± 6.67	85.7 ± 9.71	100.0 ± 0.00	*F*(3,32) = 0.21	0.885
Latency to enter successful arm (s)	27.3 ± 2.68	28.4 ± 1.76	25.0 ± 1.85	25.3 ± 2.92	*F*(3,127) = 0.96	0.415
Latency to reward (s)	32.0 ± 2.40	33.4 ± 1.73	31.6 ± 1.95	30.4 ± 3.07	*F*(3,116) = 0.37	0.772
Duration trial (s)	57.2 ± 1.78	56.6 ± 1.20	57.2 ± 1.24	59.5 ± 2.14	*F*(3,168) = 0.51	0.679
Non-compliant trials (%)	8.3 ± 2.78	5.1 ± 1.52^a^	11.6 ± 2.70^b^	7.1 ± 4.21	*F*(3,592) = 3.01	0.030
Training 2						
Number of pigs	6	14	12	6		
Number of sessions	1.9 ± 0.55	3.0 ± 0.50	1.9 ± 0.41	3.2 ± 1	*F*(3,1) = 1.46	0.532
Success rate (%)	83.1 ± 15.62	60.5 ± 14.35	87.8 ± 10.13	77.6 ± 21.65	*F*(3,1) = 0.74	0.671
Latency to enter successful arm (s)	18.4 ± 2.11	16.1 ± 1.29	16.3 ± 1.73	16.3 ± 2.29	*F*(3,67.3) = 0.38	0.765
Latency to reward (s)	22.5 ± 2.41	20.6 ± 1.66	22.2 ± 2.18	20.4 ± 2.75	*F*(3,89.1) = 0.29	0.833
Duration trial (s)	44.2 ± 2.45^a^	35.5 ± 1.69	39.9 ± 2.22	31.4 ± 2.69^b^	*F*(3,209) = 6.33	< 0.001
Non-compliant trials (%)	11.6 ± 3.14^a^	9.6 ± 1.52^a^	6.2 ± 1.71	2.8 ± 1.32^b^	*F*(3,952) = 3.31	0.020
Reversal						
Number of pigs	5	9	10	5		
Number of sessions	2.6 ± 0.73	2.9 ± 0.68	3.0 ± 0.59	2.4 ± 0.86	*F*(3,1) = 0.11	0.945
Success rate (%)	60.7 ± 22.19	33.4 ± 17.80	61.4 ± 16.57	57.1 ± 21.65	*F*(3,21) = 0.46	0.716
Latency to enter successful arm (s)	11.4 ± 2.98	18.6 ± 2.55^a^	16.8 ± 2.51	7.7 ± 3.38^b^	*F*(3,85.2) = 3.61	0.017
Latency to reward (s)	15.3 ± 3.21^ac^	19.9 ± 1.18^b^	19.1 ± 1.18^ab^	9.0 ± 1.20^c^	*F*(3,86.8) = 4.49	0.006
Duration trial (s)	34.0 ± 4.49	36.7 ± 4.01	37.5 ± 4.09	30.2 ± 4.87	*F*(3,191) = 1.1	0.352
Non-compliant trials (%)	10.6 ± 2.82	15.8 ± 2.65	15.4 ± 2.36	11.7 ± 3.11	*F*(3,802) = 0.81	0.489

Tested piglets were scored for IUGR level at birth (IUGR 0 = no sign of IUGR; IUGR 1–3 = presence of 1–3 of the characteristics for IUGR) Habituation to the experimental procedure started 3 days post-weaning (29 ± 0.3 days) and training started at approximately 37 (± 0.5) days. Training and reversal sessions lasted 60 s. During training 1 sessions, pigs were allowed to enter both choice arms to retrieve the reward (mistake allowed). In training 2 sessions, pigs were only allowed one entry attempt. In the reversal sessions, the reward arm was opposite to the one learned in training sessions, and pigs were allowed only one entry attempt

Superscript letters indicate significant differences between the IUGR levels at *P* < 0.05

^1^Numbers presented are the raw values

## Results

### T-Maze

#### Habituation and side preference

Approximately 53% of the pigs were successfully habituated to the experimental procedure. The success rate and the number of sessions to complete habituation, training (step 1 and step 2) or reversal did not differ between pigs with different neonatal supplementation (Table 1) or IUGR level (Table 2). There was an effect of the side of the reward arm on the latency to enter the reward arm in training step 1 (East 28.5 ± 1.80 s, West 24.5 ± 1.52 s; *F*_1,136_ = 4.01, *P* = 0.047) and 2 (East 19.0 ± 1.24 s, West 14.5 ± 1.32 s; *F*_1,128_ = 7.44, *P* = 0.007), and on the latency to obtain the reward in training step 2 (East 23.7 ± 1.61 s, West 19.2 ± 1.65 s; *F*_1,133_ = 6.3, *P* = 0.013) and reversal (East 20.2 ± 2.66 s, West 15.8 ± 2.49 s; *F*_1,90.7_ = 4.8, *P* = 0.031). Reward arm side also affected the duration of trial in reversal step (East 38.8 ± 4.01 s, West 30.4 ± 3.85 s; *F*_1,206_ = 15.19, *P* < 0.001).

#### Effect of neonatal supplementation

There was a tendency for an effect of neonatal supplementation on the latency to enter the reward arm in training step 1 but not in training step 2 or reversal; and there was no effect on the latency to obtain the reward at any step (Table 1). However, sham-dosed piglets had a shorter trial duration than piglets given water (*t*_208_ = − 2.69, *P* = 0.038) and piglets given a commercial product (*t*_226_ = − 2.84, *P* = 0.026) in reversal step (Table 1). Neonatal supplementation influenced the percentage of non-compliant trials in training step 1, and tended to influence the percentage of non-compliant trials in reversal step (Table 1). In training step 1, piglets given commercial product had a lower percentage of non-compliant trials than sham-dosed piglets (*t*_592_ = 2.87, *P* < 0.022). In the reversal step, piglets given coconut oil tended to have a lower percentage of non-compliant trials than piglets given water (*t*_802_ = − 2.34, *P* = 0.089).

#### Effect of IUGR level

There was an effect of IUGR level on the latency to enter the reward arm and to obtain the reward in reversal, but not in any of the training steps (Table 2). IUGR3 piglets significantly differed from IUGR1 piglets (latency arm *t*_72.8_ = 2.9, *P* = 0.024; latency reward *t*_80_ = 3.28, *P* = 0.008). They were also faster than IUGR2 piglets to obtain the reward (*t*_73.9_ = 2.77, *P* = 0.034), but only tended to be faster to enter the reward arm (*t*_70.3_ = 2.5, *P* = 0.068). Similar trends were observed between IUGR0 and IUGR1 piglets (latency arm: *t*_89.2_ = − 2.47, *P* = 0.072; latency reward: *t*_90_ = − 2.76, *P* = 0.035). There was an effect of IUGR score on the duration of trials in training step 2 (Table 2). IUGR3 piglets had shorter trials than IUGR0 piglets (*t*_229_ = 3.93, *P* < 0.001) and tended to have shorter trials than IUGR2 piglets (*t*_118_ = 2.36, *P* = 0.088). IUGR level also influenced the percentage of non-compliant trials in training step 1 and 2, but not in reversal step (Table 2). In training step 1, IUGR1 piglets had a lower percentage of non-compliant trials than IUGR2 piglets (*t*_592_ = − 3, *P* = 0.015), and in training step 2 IUGR3 piglets had a lower percentage of non-compliant trials than IUGR0 (*t*_952_ = 2.65, *P* = 0.041) and IUGR1 (*t*_952_ = 2.62, *P* = 0.044) piglets.

#### Effect of sex

Females were slower than males to enter the reward arm (29.5 ± 1.73 s vs. 23.5 ± 1.74 s, respectively; *F*_1,133_ = 7.18, *P* = 0.008) and to obtain the reward (34.6 ± 1.68 s vs. 29.1 ± 1.57 s, respectively; *F*_1,121_=5.39, *P* = 0.022), and had a higher percentage of non-compliant trials (14.6 ± 3.07% vs. 3.9 ± 1.38%, respectively; *F*_1,592_ = 10.25, *P* = 0.001) in training step 1. However, in training step 2 females had a lower percentage of non-compliant trials than males (5.2 ± 1.14% vs. 8.6 ± 1.52%, respectively; *F*_1,952_ = 3.96, *P* = 0.047); and in the reversal step, females were faster than males to enter the reward arm (11.3 ± 2.42 s vs. 16.1 ± 2.37 s, respectively; *F*_1,84.7_ = 4.22, *P* = 0.043) and to obtain the reward (15.5 ± 2.66 s vs. 20.5 ± 2.63 s, respectively; *F*_1,90.5_ = 4.66, *P* = 0.034). Females had longer trial duration than males in training step 1 (59.8 ± 1.20 s vs. 55.4 ± 1.20 s, respectively; *F*_1,179_ = 7.81, *P* = 0.006), but had a shorter trial duration in training step 2 (35.4 ± 1.73 s vs. 40.1 ± 1.70 s, respectively; *F*_1,272_ = 7.47, *P* = 0.007) and reversal (30.8 ± 3.99 s vs. 38.4 ± 3.95 s, respectively; *F*_1,191_ =  967, *P* = 0.002).

### Spontaneous Object Recognition Test (SORT)

Sex had no effect on any of the variables recorded during SORT (data not presented).

#### Interactions with object across sessions

The percentage of time interacting with both objects was 18.4% (± 2.97) on average (range 11.3–60.9%) and was not influenced by session (*F*_1,41_ = 1.31, *P* = 0.250), neonatal supplementation (*F*_3.38.2_ = 1.32, *P* = 0.282), or IUGR (*F*_2,38.6_ = 0.47, *P* = 0.628) (data not presented). Overall, the percentage of time spent interacting with the familiar object (matched for side) was lower in session 1 than in session 2 (10.9 ± 1.97 vs. 4.8 ± 1.97, respectively; *F*_1,41_ = 18.47, *P* < 0.001), but this was not affected by neonatal supplementation (S 9.7 ± 2.30, CO 10.0 ± 2.29, CP 4.8 ± 2.30, W 7.1 ± 2.43; *F*_3,38.1_ = 2.23, *P* = 0.101), or IUGR level (IUGR1 8.5 ± 2.19, IUGR2 6.8 ± 2.20, IUGR3 8.43 ± 2.33; *F*_2,38.6_ = 0.41, *P* = 0.664). However, in session 1, piglets given the commercial product spent less time interacting with the familiar object than piglets given coconut oil (*t*_76.4_ = 3.15, *P* = 0.002) and sham-dosed piglets (*t*_76.1_ = 2.56, *P* = 0.012) (Table 3).

**Table 3:** Mean (± S.E.) outcomes of the Spontaneous Object Recognition Test (SORT)

	Sham-dosed	Coconut oil	Commercial product	Water	Test statistic^2^	*P* value
Number of pigs	12	12	12	11		
Session 1						
Latency to approach objects (s)	51.1 ± 16.51	39.9 ± 16.74	67.6 ± 17.34	56.6 ± 17.68	*F*(3,133) = 0.99	0.398
Percentage of time interacting with objects (%)	5.3 ± 1.10	5.9 ± 1.10	3.4 ± 1.10	4.9 ± 1.18	*F*(3,175) = 1.56	0.201
Percentage of time interacting with familiar object (%)	13.1 ± 2.69^a^	14.8 ± 2.68^a^	5.3 ± 2.69^b^	10.6 ± 2.85	*F*(3,76.3) = 3.74	0.015
Session 2						
Latency to approach novel object (s)	30.0 ± 15.09	37.3 ± 15.10	52.5 ± 14.41	27.3 ± 15.62	*F*(3,38) = 0.59	0.628
Percentage of time interacting with familiar object (%)	6.3 ± 2.69	5.1 ± 2.68	4.2 ± 2.69	3.6 ± 2.85	*F*(3,76.3) = 0.28	0.842
Percentage of time interacting with novel object (%)	6.9 ± 1.28	7.25 ± 1.28	6.3 ± 1.28	4.8 ± 1.38	*F*(3,40) = 0.62	0.605
Percentage of pigs approaching novel object first (%)^1^	100.0 ± 0.00	72.7 ± 14.08	75.0 ± 13.06	72.7 ± 14.08	*X*^2^(3) = 3.58	0.310

Tested piglets either received a dose of energy (coconut oil or commercial product) or water (water), or were sham-dosed (sham), at 3 h post-partum. Habituation to the experimental procedure started 3 days post-weaning (29 ± 0.3 days) and pigs were tested at 41 (± 0.3) days. The two sessions lasted 5 min and were separated in time by a 15-min retention period. During session 1, pigs were exposed to two identical objects. In session 2, one object from session 1 (familiar object) was replaced by a novel object

Superscript letters indicate significant differences between the neonatal supplementations at *P* < 0.05

^1^Numbers presented are the raw values

^2^Calculated *F* values for the *F* test (GLMM), and calculated *X*^2^ values for the Wald-test (Kruskal–Wallis)

In session 1, the latency to approach the objects and the percentage of time interacting with the objects were not affected by neonatal supplementation (Table 3) or IUGR scores (Table 4). However, pigs spent a greater percentage of the session interacting with the object on the left side than the object on the right side (5.9 ± 0.94% vs. 3.9 ± 0.94%, respectively; *F*_1,174_=5.4, *P* = 0.021). Overall, in session 2, pigs approached the novel object faster than the familiar object (51.4 ± 9.61 s vs. 100.4 ± 10.61 s; *F*_1,103_ = 16.03, *P* < 0.001), and spent a greater proportion of time interacting with the novel object than with the familiar object (6.3 ± 0.67% vs. 2.3 ± 0.67%; *F*_1,173_ = 24.08, *P* < 0.001).

**Table 4:** Mean (± S.E.) outcomes of the Spontaneous Object Recognition Test (SORT)

	IUGR 1	IUGR 2	IUGR 3	Test statistic^b^	*P* value
Number of pigs	17	17	13		
Session 1					
Latency to approach objects (s)	43.9 ± 15.90	59.8 ± 15.84	57.7 ± 17.02	F(2,132) = 0.74	0.48
Percentage of time interacting with objects (%)	5.5 ± 1.02	4.0 ± 1.02	5.1 ± 1.12	F(2,169) = 1.26	0.287
Percentage of time interacting with familiar object (%)	**21.3** ± **3.98**	17.2 ± 4.00	**19.9** ± **4.39**	F(2,75.1) = 2.5	0.089
Session 2					
Latency to approach novel object (s)	37.1 ± 12.21	38.3 ± 13.08	34.9 ± 14.20	F(2,38) = 0.02	0.985
Percentage of time interacting with familiar object (%)	**20.4** ± **3.98**	16.1 ± 4.00	**15.7** ± **4.39**	F(2,75.1) = 0.38	0.683
Percentage of time interacting with novel object (%)	8.8 ± 1.08	5.1 ± 1.08	5.1 ± 1.26	F(2,40) = 3.64	0.035
Percentage of pigs approaching novel object first (%)^a^	94.1 ± 5.88	80.0 ± 10.69	61.5 ± 14.04	*X*^2^(2) = 4.78	0.092

Tested piglets were scored for IUGR level at birth (IUGR 0 = no sign of IUGR; IUGR 1 to 3 = presence of 1 to 3 for the characteristics for IUGR). Habituation to the experimental procedure started 3 days post-weaning (29 ± 0.3 days) and pigs were tested at 41 (± 0.3) days. The two sessions lasted 5 min and were separated in time by a 15-min retention period. During session 1, pigs were exposed to two identical objects. In session 2, one object from session 1 (familiar object) was replaced by a novel object.

Values in bold indicate significant differences between the sessions, within the same IUGR level (*P* < 0.05)

^a^Numbers presented are the raw values

^b^Calculated *F* values for the *F* test (GLMM), and calculated *X*^2^ values for the Wald-test (Kruskal–Wallis)

#### Effect of neonatal supplementation

**Fig. 2:**
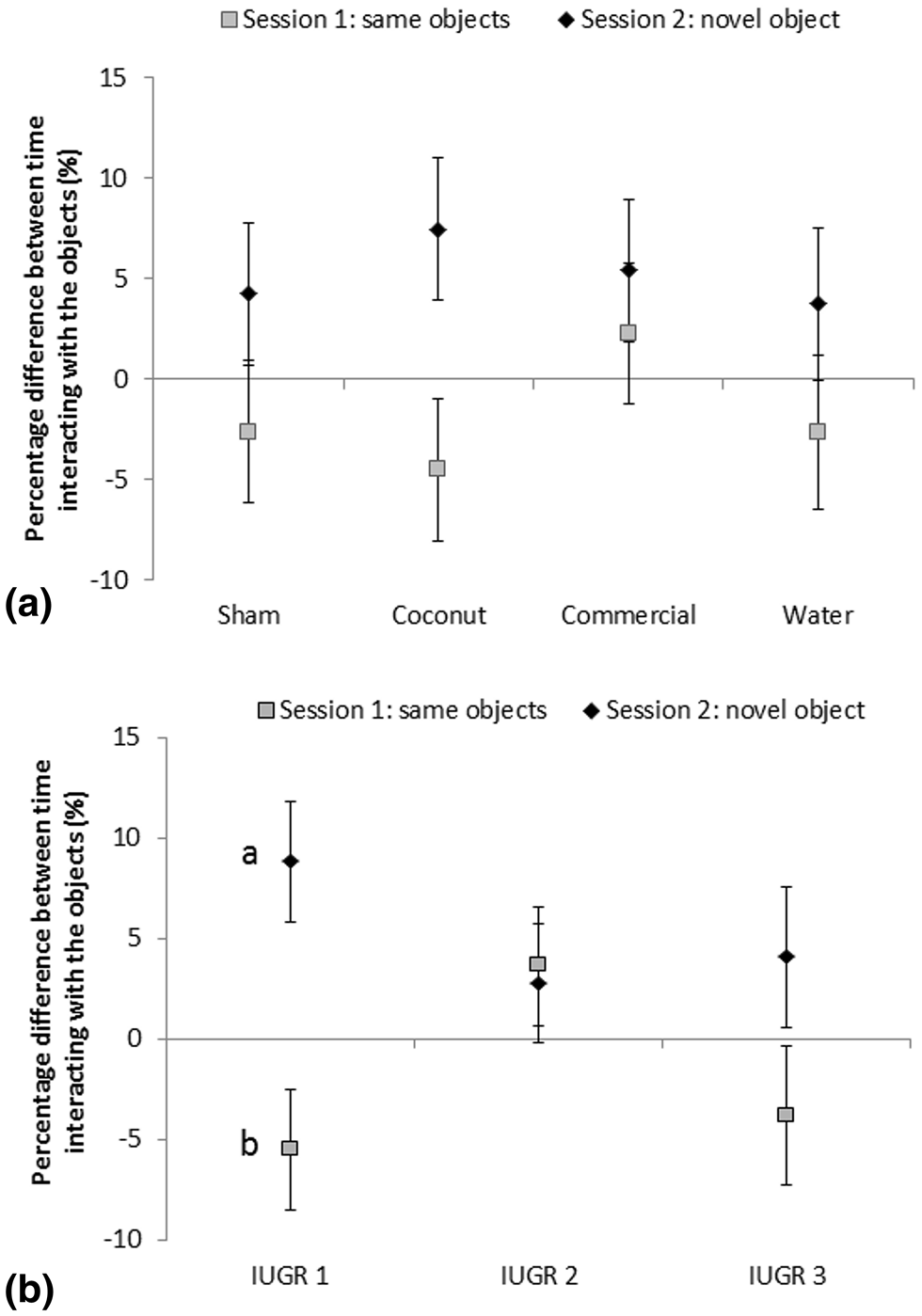
Mean (± S.E.) Percentage difference between time spent interacting with the objects in the Spontaneous Object Recognition Test (approximately 41 days). Sessions were separated by a 15-min retention time. Superscript letters indicate differences between the two sessions within one category of pigs (^a,b^*P* < 0.05): **a** Different neonatal supplementation of the pigs (energy: coconut oil or commercial product; no energy: water or sham-dosed). Effects: supplementation: *P* = 0.788; session: *P* = 0.007; supplementation × session: *P* = 0.660. **b** Different levels of IUGR at birth (IUGR 1–3 = presence of 1–3 for the characteristics for IUGR). Effects: IUGR: *P* = 0.638; session: *P* = 0.007; IUGR × session: *P* = 0.042

Neonatal supplementation did not influence the latency to approach the novel object (*F*_3,38_ = 0.59, *P* > 0.628), the percentage of time interacting with it (*F*_3,40_ = 0.62, *P* = 0.605), the percentage of pigs choosing to approach the novel object first (*X*^2^_3_ = 3.58, *P* = 0.310) (Table 3), or the percentage difference in time spent interacting with the novel and familiar objects (*F*_3,81_ = 0.35, *P* = 0.788; Fig. 2a).

#### Effect of IUGR level

There was no significant effect of IUGR level on the latency to approach the novel object (*F*_2,38_ = 0.02, *P* = 0.985; Table 4). However, IUGR level affected the percentage of time interacting with the novel object (*F*_2,40_ = 3.64, *P* = 0.035; Table 4), as IUGR1 pigs tended to interact more with the novel object than IUGR2 (*t*_40_ = 2.41, *P* = 0.053) and IUGR3 (*t*_40_ = 2.19, *P* = 0.085) pigs. The percentage of pigs choosing to approach the novel object first tended to be affected by IUGR level (*X*^2^_2_ = 4.78, *P* = 0.092), since there was a tendency for a greater percentage of IUGR1 pigs approaching the novel object first, compared to IUGR3 pigs (DSCF-value = 3.07; *P* = 0.076). There was no effect of IUGR level on the percentage difference in time spent interacting with the novel and familiar objects (*F*_2,81_ = 0.45, *P* = 0.638), but the interaction between IUGR and session was significant (*F*_2,81_ = 3.29, *P* = 0.042; Fig. 2b).

## Discussion

This study aimed to investigate the effects of IUGR levels and neonatal supplementation on the post-weaning cognitive abilities of low birth-weight pigs in a T-maze task and in a Spontaneous Object Recognition Test. Together, the results suggest that some low birth-weight pigs, independent of their level of IUGR or neonatal supplementation, are able to learn a spatial task and to discriminate between a novel object and a familiar object. Some performance indicators in the T-Maze task and SORT were modulated by IUGR level of the piglets, but not by neonatal supplementation.

Only approximately half of the pigs could be habituated to the T-Maze task, although the habituation protocol followed the recommendation of Elmore et al. (2012). Failure to habituate to the experimental procedure implies a failure to cope with the associated stressors (e.g., social isolation, presence of human, movement of doors). A recent study by Vazquez-Gomez et al. (2016) demonstrated that low birth-weight piglets had lower concentrations of catecholamine neurotransmitters, which are related to learning and memory abilities, reward-motivated behaviour and stress. In addition, Antonides et al. (2012) suggested that low birth-weight piglets might have a greater emotional reactivity than normal birth-weight piglets. Together, these findings could explain the poor success rate of habituation of the present study, as low birth-weight piglets may be more susceptible to stress, and may not have coped with the stressors associated with the testing procedure (e.g., social isolation, lifting of the guillotine door) or may have had a lower food motivation. Unfortunately, cognition studies rarely mention the habituation success of their test procedures, which makes comparisons and optimisation difficult. The large drop-out in the habituation and training phases of the present study resulted in an unbalanced dataset, which could have potentially biased results of the T-maze test despite attempting to account for the unbalanced numbers in the statistical analysis. In particular, the effect of the side of the reward arm in training step 2 and reversal step is likely to be due to a lack of control over the pigs’ progress in completing the task (i.e., all phases of the test).

The T-Maze task was validated in pigs by comparing control pigs (administered with saline) with pigs which were administered with scopolamine, which impaired their spatial learning abilities (Elmore et al. 2012). The test was then applied to experimental populations of pigs selected to be extreme with either mild or severe iron deficiency (no injection of iron at birth, and fed a mildly or severely iron-deficient feed; Rytych et al. 2012), which showed impaired performances compared to control pigs. Therefore, it can be hypothesised that any difference between IUGR levels or neonatal energy supplementations in the present study may be more subtle to detect. Piglets with the most severe symptoms of IUGR at birth (IUGR3) had the best performances (shortest latency to enter the reward arm and obtain the reward) in the reversal step, suggesting that they may be more flexible in their learning. They also had a lower proportion of non-compliant trials during the training step 2, compared to pigs with low levels of IUGR (IUGR0 and IUGR1), which indicate better coping with the training procedure. Indeed, the switch from free exploration in step 1 to only one entry allowed in step 2 could be indicative of frustration for the pigs and those failing to cope with may be the result of loss in interest/motivation for the test. The brain-sparing effect, by ensuring correct development of the brain, could explain the better performance of piglets with severe IUGR level. However, this would also suggest that brain-sparing only occurs at a certain threshold of growth restriction, thus piglets with intermediate levels of IUGR may not benefit fully from this phenomenon. Another possible explanation would be that piglets with severe IUGR level have lower chances of survival and thus would have greater learning and adaptive capacities than piglets with higher chances of survival. Antonides et al. (2015a) also discuss these theories regarding their finding that (very) low birth-weight piglets performed better in a hole-board task than normal birth-weight piglets.

During the first session of SORT, where two identical objects were presented, there seemed to be a bias in side preference of the pigs as, overall, they spent more time interacting with the left object than the right object. The study of Antonides et al. (2012) suggested that low birth-weight piglets might be more emotionally reactive than normal birth-weight piglets in a situation where they are socially isolated. Therefore, when the two objects were identical, test pigs might feel more comfortable interacting with the left object as it was closer to the pen containing the companion pigs, which the test pig could hear.

The reduction in the percentage of time interacting with the familiar object showed pigs’ habituation to this over the two sessions, which is in accordance with the expectation of the test (Moustgaard et al. 2002). The latency to approach the novel object was not affected by either neonatal supplementation or IUGR level, demonstrating that these factors did not affect the initial reaction to a novel object. When it came to the difference in time spent interacting with the novel and familiar objects, overall, low birth-weight piglets seemed able to discriminate between the novel and familiar objects. Similar to the latency to approach, this was not affected by neonatal supplementation or IUGR level. However, only IUGR1 piglets had a significant difference between sessions in the difference in time spent interacting with the novel and familiar objects. This insinuates that pigs with IUGR, and especially IUGR3 pigs, might not be as capable in discriminating between the objects as piglets without IUGR, or might not show a preference towards novelty. Although there were no significant differences, there was a tendency for fewer IUGR3 pigs to approach the novel object first and to interact with it less, compared to IUGR1 pigs, which further suggests that piglets with severe IUGR at birth might be less attracted to (or more fearful of) novelty, or failed to discriminate between the objects.

Our results also indicate that female pigs performed better than males in the T-maze test but not in the SORT, and thus gender may not influence all types of cognitive abilities (Kornum and Knudsen 2011). The absence of sex effect on the performance of pigs in SORT could be due to the fact that the SORT is less demanding in terms of memorisation. Indeed, in the SORT a piglet’s memory was tested over a short amount of time (retention time = 15 min), while in the T-maze task piglets had to memorise the reward’s location between testing sessions (retention time = 24 h). Martin et al. (2015) found that male pigs interacted more with the novel object than female pigs. However, considering other results of the study, the authors suggested that this difference was more likely related to motivation to play than cognitive performance. Roelofs et al. (2017) demonstrated that normal birth-weight male and female pigs had similar learning performance in the initial learning phase of a hole-board spatial task, but females were faster to retrieve rewards in reversal phase, which suggests a more flexible response to reversed learning. Similarly, in the present study females had a lower performance than males in training step 1 (slower to enter the reward arm and to obtain the reward, higher percentage of non-compliant trials) but they outperformed males in the reversal step (faster to enter the reward arm and to obtain the reward). There are contradicting results on the effect of gender on cognitive performance (Roelofs et al. 2017, 2018) that could be related to stress levels and housing conditions (Roelofs et al. 2018). Given the small sample size, the present study should be considered an exploratory investigation which highlights the importance of assessing piglet IUGR level in cognitive studies. Our results suggest that IUGR level has a different effect on pigs’ cognitive abilities, as pigs with severe levels of IUGR appeared better at reversing their learning (behavioural flexibility) but may have impaired abilities to discriminate between a novel and familiar object (short-term memory). Further work is needed to validate the present results and to explore factors influencing the development of cognitive abilities in low birth-weight piglets, such as their capacity to recover physically (e.g., compensatory growth) from IUGR during lactation (Amdi et al. 2015).

## Conclusion

This work shows that some low birth-weight piglets are able to discriminate between a novel and a familiar object, and to successfully complete a spatial learning task. The results also suggest that there might be subtle differences between piglets with different levels of IUGR. However, given the small sample size of the present study, results should be taken with caution and further work should be carried out to address this hypothesis.
